# Effects of lifestyle intervention and supplementation with insoluble oat fiber on cognitive functions in patients with prediabetes: a secondary analysis of the Optimal Fiber Trial

**DOI:** 10.3389/fnut.2025.1699958

**Published:** 2026-01-16

**Authors:** Stefan Kabisch, Federico Montagna, Caroline Honsek, Margrit Kemper, Christiana Gerbracht, Ayman M. Arafat, Andreas L. Birkenfeld, Ulrike Dambeck, Martin A. Osterhoff, Martin O. Weickert, Agnes Flöel, Andreas F. H. Pfeiffer

**Affiliations:** 1 Department of Endocrinology and Metabolic Medicine, Campus Benjamin Franklin, Charité University Medicine, Berlin, Germany; 2 Deutsches Zentrum für Diabetesforschung e.V., Geschäftsstelle am Helmholtz-Zentrum München, Neuherberg, Germany; 3 Department of Clinical Nutrition, German Institute of Human Nutrition Potsdam-Rehbruecke, Nuthetal, Germany; 4 Division of Diabetology, Endocrinology and Nephrology, Department of Internal Medicine IV, Eberhard-Karls University Tübingen, Tübingen, Germany; 5 Institute for Diabetes Research and Metabolic Diseases of the Helmholtz Center Munich at the University of Tübingen, Tübingen, Germany; 6 Warwickshire Institute for the Study of Diabetes, Endocrinology and Metabolism, Coventry, United Kingdom; 7 The ARDEN NET Centre, ENETS CoE, Coventry, United Kingdom; 8 University Hospitals Coventry and Warwickshire NHS Trust, Coventry, United Kingdom; 9 Centre of Applied Biological and Exercise Sciences (ABES), Faculty of Health and Life Sciences, Coventry University, Coventry, United Kingdom; 10 Division of Biomedical Sciences, Department of Translational and Experimental Medicine, Warwick Medical School, University of Warwick, Coventry, United Kingdom; 11 Department of Neurology, University Medicine Greifswald, Greifswald, Germany; 12 German Center for Neurodegenerative Diseases, Standort Rostock/Greifswald, Germany

**Keywords:** prediabetes, diabetes prevention, insoluble fiber, memory, cognition, learning, impaired glucose tolerance, type 2 diabetes

## Abstract

**Background:**

In cohort studies, intake of insoluble cereal fiber is associated with multiple health benefits, including preserved cognitive functions. However, evidence from intervention studies is sparse. In the Optimal Fiber Trial (OptiFiT), lifestyle changes and supplementation with oat fiber in prediabetes patients improved glycemic metabolism and body composition, which could be linked to cognitive changes.

**Methods:**

In OptiFiT, 180 patients with impaired glucose tolerance received either an insoluble fiber supplement or a placebo for 2 years in a double-blind, randomized approach, and underwent a parallel 1-year complex lifestyle intervention program. Annual visits included metabolic, anthropometric, and cognitive assessments: Mini-Mental State Examination (MMSE), Verbal Learning Memory Test (VLMT), Regensburg Word Fluency Test (RWFT), Number Connection Test (NCT), Number Recall Test (NRT), and Rey–Osterrieth Complex Figure Test (RCFT). Group-wise comparisons were conducted both globally as well as stratified by age.

**Results:**

Cognitive functions only slightly improved—particularly in VLMT and RWFT—without major differences by group or age. At baseline, cognitive function measured by RCFT recall, VLMT, RWFT, and backwards NRT was inversely correlated with age, but not with HbA1c, fasting, or postprandial glucose levels.

**Conclusion:**

Beneficial effects of insoluble fiber and lifestyle intervention on glycemia might not translate into preserved cognitive capabilities in middle-to-higher aged patients with prediabetes in a 2-year intervention period. Long-term intervention studies in patients with both cognitive vulnerability and metabolic susceptibility are warranted. Such large RCTs should also corroborate putatively involved mechanisms in the epidemiologically assumed protection from cognitive decline.

**Clinical trial registration:**

Clinicaltrials.gov, identifier NCT 01681173.

## Introduction

Type 2 diabetes mellitus (T2DM) contributes to a smorgasbord of long-term complications, thus being a major burden for individual patients, the healthcare system, and the entire society. As one late outcome, cognitive decline and dementia need to be considered (1). Despite great efforts for prevention, prevalences for T2DM and prediabetes are still rising all over the world. A large proportion of cases could be avoided by suitable measures targeting the pivotal lifestyle of energy-dense (mal)nutrition and lack of physical activity (2–5). Prediabetes and T2DM are strongly linked to (visceral) obesity and NAFLD (6–8), leading to the encouragement of weight loss as a major treatment component. However, diabetes prevention and remission by plain weight loss might be counterbalanced by poor compliance or side effects, particularly for cognitive capabilities (9–11).

Specific dietary aspects of a protective, healthy diet beyond caloric restriction include limited intake of saturated fats, sugars, and alcohol, while enforcing the consumption of fiber-rich foods (12). However, behavioral restraints, time issues, and limited affordability drastically reduce long-term compliance, almost irrespective of the overall dietary pattern (13). Additionally, for most dietary components, evidence for preventive effects is mainly of an epidemiological nature and has been partially disputed for decades. For insoluble cereal fiber, very few RCTs in humans exist, even though cohort studies imply strong risk reductions for T2DM (14), NAFLD (15, 16), and cardiovascular disease (17). For risk of dementia, high GI and highly processed foods are deemed detrimental (18–20), while vegetables and other sources of fiber are considered beneficial (21).

In RCTs, complex lifestyle interventions improved or preserved cognitive functions, while attributing effects to specific lifestyle components is impossible herein (22, 23). In large-scale dietary RCTs, the Mediterranean diet (24), but not the low-fat diet or the DASH diet, proved effective to preserve and/or improve memory and cognition (25–27). Overall, mild impairments in middle-aged persons seem to be treatable, while older patients do not benefit (28–32). RCTs on specific foods and their components are sparse. Studies comparing high- vs. low-GI foods—done in children or younger healthy adults—mostly failed to show a cognitive benefit (33–35). Intervention with fiber-rich grains was able to improve cognitive parameters in some (36–38), but not all studies (39). Few trials on polydextrose, mixed grain, Jabocitaba peel, guar gum, or polyphenol- and fiber-rich fruit extracts revealed inconsistently positive effects in healthy persons from adolescence to late adulthood (40–45). All mentioned studies differed considerably in the dosage and type of fiber, duration of intervention, and targeted cohort. In general, persons during adolescence and at older age might be promising cohorts for investigations of food-related effects on cognition.

As insoluble fiber shows more promising protective associations with a wide range of long-term outcomes in cohort studies, our focus in a series of RCTs was set on that particular group of fiber. Under the assumption that insoluble cereal fiber improves glycemia, inflammation, and vascular health, long-term benefits on cognition could be expected. Those short-term improvements could be driven by mechanisms related to gut microbiota, incretins, bile acids, or modulation of the mTOR pathway. The Protein, Fiber, and Metabolic Syndrome study and the Optimal Fiber Trial (OptiFiT) both investigated those mechanisms and showed dose-dependent fiber-driven improvements of insulin resistance, glucose metabolism, and inflammation (46–48), particularly in patients with NAFLD-linked prediabetes (49), and mainly independent from weight loss.

OptiFiT, as a 2-year RCT, also assessed cognitive functions and might thereby provide novel data on the impact of insoluble oat fiber on memory in elderly persons with metabolic impairment.

## Research design and methods

For OptiFiT, the core paper published elsewhere documented ethics approval, study registration and recruitment, inclusion and exclusion criteria, and the overall study design (47). Shortly, we recruited 180 subjects with impaired glucose tolerance (IGT), a metabolic subtype with high risk for progression to T2DM (50). Impaired glucose tolerance was diagnosed by capillary blood glucose measurement (140–200 mg/dL) at the 2-h time point of a 75-gram oral Glucose Tolerance Test. Patients were eligible with an age of at least 18 years and absence of severe and/or untreated internistic or psychiatric disorder. Annual metabolic measurements encompassed fasting blood sampling, oral glucose tolerance tests, anthropometrics (47), and a cognitive test battery, given in more detail below.

The intervention entailed 2-year supplementation with a blinded drinking supplement providing fiber or placebo and an overlapping lifestyle group consultation during the first 12 months of supplementation, based on the prevention concept PREDIAS (51). Those consultations focused on increased physical activity (30 min/day), reduced intake of total fat (<30 kcal%), and saturated fat (<10 kcal%), as well as increased intake of total dietary fiber (>15 g/1000 kcal). Dietary baseline status and interventional compliance were assessed by 4-day food records, which were analyzed using the nutrition software PRODI® 5.8 based on Bundeslebensmittelschlüssel 3.0 (52). The fiber supplement provided a daily dose of 15 g of poorly fermentable, insoluble oat fiber (~70% cellulose, ~25% hemicellulose; ~3–5% lignin), while the main component of the placebo was the slowly digestible disaccharide isomaltulose. Both drinking powders were similar in taste, odor, smell, and appearance. Details on the double-blinded fiber supplement and adherence control are published in the core paper (47).

### Cognitive assessment

During the annual visits, cognitive tests were conducted in the following fashion. Patients started with the Mini-Mental State Examination (MMSE), entailing various tasks on short-term memory and awareness (53). The Rey–Osterrieth Complex Figure Test (RCFT) was presented as a template to be copied by hand drawing (54, 55). For the Verbal Learning Memory Test (VLMT), randomly chosen lists of words were read to the patients to be repeated instantly in sequential attempts. Interference cues increase the challenge of proper recollection (56). The Regensburg Word Fluency Test (RWFT) enforces imagining phonems or semantic items (e.g., animals and objects) with a given starting letter within 60 s of time. Initial letters and semantic groups were randomized (57). For the number connection test (NCT), a connecting line between incremental sets of randomly dispersed numbers (and letters) has to be drawn as quickly as possible; the required time was assessed (58, 59). In the number recall test (NRT), patients were asked to repeat sets of increasingly longer numbers. After a first set using the same order, the patients had to repeat the next numbers in the opposite order (60, 61). A concluding second run of the RCFT entailed recalling the complex figure without a template.

### Statistical analyses

This analysis was done by intention-to-treat principles; missing data were filled by the last-observation-carried-forward method. Kolmogorov–Smirnov tests showed frequent absence of normal distribution; we therefore used Mann–Whitney and Wilcoxon tests for between-group and within-group comparisons, respectively. Stratified analyses by age (<60 years vs. ≥60 years) were also conducted in the same way. Spearman correlations were used to assess the interaction of age, glycemia, and cognition at baseline and over time. All data are presented as means ± standard deviation. The results were considered significantly different if *p* < 0.05. All statistical analyses were performed using the SPSS for Windows program version 26.0 (SPSS Inc., Chicago, IL, USA).

## Results

### General cohort description

Major anthropometric and metabolic baseline data for this cohort are given in Table 1. Besides a slightly skewed sex distribution, there are no differences between the fiber and placebo groups. A full description of further metabolic parameters and the baseline dietary profile can be found elsewhere. Here, too, we have already shown that increased fiber intake could only significantly be achieved by the fiber supplement (+15 grams per day), while lifestyle changes accounted for an increase of <1 gram per day (47).

**Table 1:** Anthropometric and metabolic baseline data.

Parameter	Total	Fiber	Placebo	*p*-value
Patient count (n)	180	89	91	
Sex (female; %, n)	66.6% (120)	74.2% (64)	59.3% (53)	0.040*
Age (years)	60 ± 10	59 ± 10	60 ± 10	0,729
BMI (kg/m^2^)	32.4 ± 5.9	31.8 ± 5.3	33.0 ± 6.4	0,483
Fasting glucose (mg/dl)	107.1 ± 11.7	106.9 ± 11,8	107.4 ± 11.7	0,957
2-h glucose oGTT (mg/dl)	163.8 ± 30.7	162.6 ± 29.8	164.9 ± 31.7	0,628
HbA1c (%)	5.6 ± 0.4	5.7 ± 0.4	5.6 ± 0.4	0,475

Baseline data; means and SD; comparison by Mann–Whitney U-tests and X^2^ tests; *p < 0.05.

### Baseline description of cognitive state

For baseline, measures of cognitive functions are reported in Table 2. Baseline MMSE, on average, did not indicate cognitive dysfunction in our cohort. Only 10 out of 180 patients had an MMSE score below 27 (all of which were above 23 points), indicating very minor cognitive dysfunction. In the RCFT, copying the figure with the template present was uncomplicated for almost all patients; 11 out of 180 patients had only 29–32 points (out of a total of 36). Template-free recall of the figure after an interspersed period of other cognitive tests resulted in a more widely distributed range of results, with an average of 16 points below the initial test result. In the VLMT, consecutive attempts of word recall resulted in improved results, as expected. The interference list typically impaired recollection of the initial word list, and subsequent tests showed the canonical re-improvement. Both the NCT and the RWFT were completed with, on average, normal results, but a broader range of values. The NRT did not show severe impairments of short-term memory. Baseline comparison between the groups did not reveal major differences, with only the placebo group showing better results in the RCFT recall.

**Table 2:** Cognitive outcomes over 2 years, total cohort.

Cognitive test	Fiber			Placebo			*p*-value		
	Baseline	1 year	2 years	Baseline	1 year	2 years	Baseline	1 year	2 years
MMSE (pts.)	28 ± 1	0 ± 1	0 ± 1	28 ± 1	0 ± 1	0 ± 1	0.781	0.837	0.236
RCFT									
Copying (pts.)	35 ± 1	0 ± 2	-1 ± 5	35 ± 1	-1 ± 4	-1 ± 5	0.545	0.461	0.827
Recall (pts.)	19 ± 6	2 ± 5**	3 ± 6**	21 ± 5	1 ± 5	0 ± 4	0.005**	0.423	0.029*
VLMT									
1st attempt (pts.)	6 ± 2	0 ± 2*	0 ± 2**	6 ± 1	0 ± 2	0 ± 2*	0.538	0.201	0.997
2nd attempt (pts.)	8 ± 2	1 ± 2**	1 ± 2**	8 ± 2	0 ± 2*	1 ± 2**	0.703	0.060	0.845
3rd attempt (pts.)	10 ± 2	0 ± 2**	0 ± 2*	10 ± 2	0 ± 3	1 ± 2*	0.463	0.149	0.595
4th attempt (pts.)	10 ± 2	1 ± 2**	1 ± 2**	11 ± 2	0 ± 3	0 ± 2*	0.256	0.043*	0.311
5th attempt (pts.)	11 ± 2	0 ± 2	0 ± 2*	11 ± 2	0 ± 3	0 ± 2**	0.616	0.711	0.377
Interference list (pts.)	5 ± 2	0 ± 2	0 ± 2*	5 ± 2	0 ± 1	0 ± 2	0.952	0.826	0.952
6th attempt (pts.)	9 ± 3	1 ± 2**	0 ± 2	9 ± 2	0 ± 2	0 ± 2	0.877	0.128	0.626
7th attempt (pts.)	10 ± 2	1 ± 2**	0 ± 2	10 ± 3	0 ± 3	0 ± 3	0.518	0.130	0.966
Recall list (pts.)	13 ± 2	0 ± 2	0 ± 2	13 ± 2	0 ± 2*	0 ± 3	0.928	0.529	0.807
Recall interference (pts.)	13 ± 2	0 ± 2*	0 ± 3	13 ± 2	0 ± 2	0 ± 2	0.276	0.043*	0.252
Full recall (pts.)	18 ± 2	0 ± 3*	0 ± 4	18 ± 2	0 ± 2	0 ± 3	0.812	0.678	0.804
NCT									
Numbers (sec)	41 ± 13	0 ± 16	5 ± 14	39 ± 20	-2 ± 17	-4 ± 20*	0.132	0.701	0.240
Numbers and letters (sec)	93 ± 41	1 ± 37	0 ± 35	84 ± 35	-4 ± 37	−8 ± 32	0.154	0.324	0.374
RWFT									
Phonemes, 1st attempt (pts.)	15 ± 4	5 ± 4	0 ± 4	15 ± 4	0 ± 4	0 ± 4	0.868	0.491	0.594
Phonemes, 2nd attempt (pts.)	11 ± 4	1 ± 4**	0 ± 4*	11 ± 4	0 ± 5	1 ± 4*	0.789	0.175	0.067
Semantic, 1st attempt (pts.)	19 ± 5	0 ± 9	−1 ± 6	20 ± 6	0 ± 8	−4 ± 6	0.459	0.690	0.100
Semantic, 2nd attempt (pts.)	12 ± 4	0 ± 3	0 ± 4	12 ± 4	1 ± 4*	0 ± 4*	0.800	0.107	0.927
NRT									
Ahead (pts.)	7 ± 1	0 ± 1	0 ± 1	7 ± 2	0 ± 1	0 ± 1	0.898	0.711	0.278
Backwards (pts.)	5 ± 1	0 ± 1	0 ± 1	5 ± 1	0 ± 1*	0 ± 1	0.320	0.084	0.768
Total (pts.)	13 ± 2	0 ± 2	0 ± 2	13 ± 3	0 ± 2	0 ± 2	0.989	0.309	0.247

Results of the cognitive tests; total cohort; baseline values and changes over time; means and SD; within-group and between-group comparisons with Wilcoxon tests and Mann–Whitney U-tests; *p < 0.05; **p < 0.01. MMSE, mini-mental state examination; NCT, number-connection test; NRT: number-recall test; RCFT, Rey–Osterrieth complex figure test; RWFT: Regensburg word fluency test; VLMT, verbal learning memory test. For MMSE, RCFT, VLMT, RWFT, and NRT, increases are improvements, while for the NCT, decreases indicate improvement.

### Changes of cognitive state in the entire cohort

The 1- and 2-year changes were limited. Significant improvements in the fiber group are found for the RCFT recall, most attempts of the VLMT, and spuriously within the RWFT. The placebo group showed improvements in the VLMT, the NCT, the NRT, and the RWFT, predominantly after 2 years. Differences between changes within the groups occurred in the RCFT recall (after 2 years), as well as the fourth attempt of VLMT and the VLMT recall interference scale (both after one year, only), with all three differences favoring the fiber group (Table 2).

### Changes of cognitive state in age subgroups

We then split the cohort by age, using a cut-off of 60 years. In younger patients, overall, the 1- and 2-year changes were once again small. Significant within-group improvements are found in the fiber group for the RCFT recall test after 2 years, as well as several attempts of the VLMT and one attempt of the RWFT, all during both intervention years. The placebo group improved spuriously in the VLMT, the NCT, and the RWFT, and contradictorily impaired in another RWFT sub-scale. Significant effects of the placebo group were mostly found after 2 years, only. The placebo and fiber groups did not differ in their 1- or 2-year outcome of any test (Table 3).

**Table 3:** Cognitive outcomes over 2 years, younger patients (<60 years of age).

Cognitive test	Fiber			Placebo			*p*-value		
	Baseline	1 year	2 years	Baseline	1 year	2 years	Baseline	1 year	2 years
MMSE (pts.)	28 ± 1	0 ± 1	0 ± 1	28 ± 1	0 ± 1	0 ± 1	0.851	0.867	0.562
RCFT									
Copying (pts.)	35 ± 1	0 ± 1	0 ± 7	35 ± 1	0 ± 1	−1 ± 7	0.563	0.762	0.530
Recall (pts.)	19 ± 6	1 ± 7	5 ± 6**	23 ± 5	1 ± 5	1 ± 4	0.010**	0.739	0.780
VLMT									
1st attempt (pts.)	6 ± 1	0 ± 1	0 ± 1	6 ± 1	0 ± 1	1 ± 2	0.329	0.455	0.639
2nd attempt (pts.)	9 ± 2	1 ± 2*	1 ± 2*	9 ± 1	0 ± 2	1 ± 2*	0.840	0.914	0.725
3rd attempt (pts.)	10 ± 1	1 ± 1**	1 ± 1**	10 ± 2	0 ± 3	1 ± 3	0.978	0.960	0.733
4th attempt (pts.)	11 ± 2	1 ± 3**	1 ± 3**	11 ± 2	0 ± 3	0 ± 2	0.218	0.939	0.645
5th attempt (pts.)	11 ± 2	0 ± 2	1 ± 3	12 ± 1	0 ± 3	0 ± 2	0.625	0.824	0.871
Interference list (pts.)	6 ± 2	0 ± 2	0 ± 1	6 ± 2	0 ± 2	0 ± 2	0.815	0.665	0.543
6th attempt (pts.)	10 ± 3	0 ± 2	0 ± 2	10 ± 2	0 ± 2*	1 ± 2	0.311	0.294	0.332
7th attempt (pts.)	11 ± 2	0 ± 1*	0 ± 2	10 ± 3	0 ± 2	0 ± 3	0.558	0.940	0.383
Recall list (pts.)	13 ± 1	0 ± 1	0 ± 1	13 ± 2	0 ± 2	0 ± 4	0.897	0.269	0.355
Recall interference (pts.)	14 ± 1	0 ± 2	0 ± 1	14 ± 2	0 ± 2	0 ± 3	0.679	0.504	0.793
Full recall (pts.)	19 ± 0	0 ± 1	0 ± 0	19 ± 3	0 ± 3	0 ± 4	0.904	0.519	0.347
NCT									
Numbers (sec)	36 ± 10	0 ± 16	−3 ± 9	34 ± 18	−4 ± 21	−5 ± 27*	0.150	0.292	0.629
Numbers and letters (sec)	78 ± 28	2 ± 32	1 ± 37	70 ± 31	−5 ± 45	−5 ± 23	0.226	0.577	0.946
RWFT									
Phonemes, 1st attempt (pts.)	14 ± 5	1 ± 4	0 ± 4	15 ± 4	0 ± 4	0 ± 4	0.357	0.836	0.747
Phonemes, 2nd attempt (pts.)	10 ± 4	1 ± 3*	1 ± 3*	12 ± 4	0 ± 5	1 ± 4	0.304	0.593	0.669
Semantic, 1st attempt (pts.)	19 ± 5	0 ± 8	0 ± 6	20 ± 7	1 ± 9	−4 ± 4*	0.752	0.238	0.526
Semantic, 2nd attempt (pts.)	12 ± 4	0 ± 3	0 ± 3	13 ± 4	1 ± 4	1 ± 3*	0.156	0.786	0.463
NRT									
Ahead (pts.)	7 ± 1	0 ± 1	0 ± 1	7 ± 2	0 ± 1	0 ± 1	0.937	0.299	0.811
Backwards (pts.)	5 ± 1	0 ± 1	0 ± 1	6 ± 1	0 ± 1	0 ± 2	0.134	0.479	0.594
Total (pts.)	13 ± 3	0 ± 1	0 ± 2	14 ± 3	0 ± 2	0 ± 2	0.444	0.675	0.628

Results of the cognitive tests; patients <60 years of age; baseline values and changes over time; means and SD; within-group and between-group comparisons with Wilcoxon tests and Mann–Whitney U-tests; *p < 0.05; **p < 0.01. MMSE, mini-mental state examination; NCT, number-connection test; NRT, number-recall test; RCFT, Rey–Osterrieth complex Figure test; RWFT: Regensburg word fluency test; VLMT: verbal learning memory test. For MMSE, RCFT, VLMT, RWFT, and NRT, increases are improvements, while for the NCT, decreases indicate improvement.

In older patients, 1- and 2-year changes were minute as well. Significant within-group 1-year improvements and 2-year impairments are found in the fiber group for the RCFT and some sub-scales of VLMT and RWFT. In the placebo group, VLMT, RWFT, and NRT show significant beneficial and detrimental changes. Outcomes differed between the fiber and placebo groups in an inconsistent pattern, with superiority of either group in various test scales, mostly after 2 years of intervention (Table 4).

**Table 4:** Cognitive outcomes over 2 years, older patients (≥60 years of age).

Cognitive test	Fiber			Placebo			*p*-value		
	Baseline	1 year	2 years	Baseline	1 year	2 years	Baseline	1 year	2 years
MMSE (pts.)	28 ± 1	0 ± 1	0 ± 1	28 ± 1	0 ± 1	0 ± 1	0.856	0.880	0.783
RCFT									
Copying (pts.)	35 ± 1	0 ± 2*	−1 ± 2*	35 ± 1	−1 ± 6	0 ± 2	0.718	0.070	0.035*
Recall (pts.)	18 ± 6	2 ± 4**	1 ± 6	20 ± 5	1 ± 6	0 ± 4	0.116	0.457	0.044*
VLMT									
1st attempt (pts.)	5 ± 2	1 ± 2*	0 ± 2*	5 ± 1	0 ± 2	0 ± 2	0.905	0.175	0.357
2nd attempt (pts.)	8 ± 2	1 ± 2**	0 ± 2**	8 ± 2	0 ± 1	0 ± 2*	0.380	0.558	0.853
3rd attempt (pts.)	10 ± 2	0 ± 2	0 ± 2	9 ± 2	0 ± 2	1 ± 2	0.361	0.147	0.011*
4th attempt (pts.)	10 ± 2	0 ± 2	0 ± 2	10 ± 2	0 ± 2	1 ± 2	0.687	0.114	0.580
5th attempt (pts.)	10 ± 3	0 ± 2	0 ± 2	11 ± 2	0 ± 3	0 ± 2*	0.867	0.595	0.605
Interference list (pts.)	5 ± 2	0 ± 1**	0 ± 1**	5 ± 1	0 ± 1	0 ± 1*	0.884	0.013*	0.037*
6th attempt (pts.)	8 ± 2	1 ± 2**	0 ± 2	9 ± 3	0 ± 2	0 ± 2	0.433	0.209	0.853
7th attempt (pts.)	8 ± 2	1 ± 2**	0 ± 2	9 ± 3	0 ± 3	0 ± 3	0.222	0.136	0.901
Recall list (pts.)	13 ± 2	0 ± 2	0 ± 3	12 ± 1	0 ± 2*	0 ± 1	0.748	0.243	0.702
Recall interference (pts.)	13 ± 2	1 ± 3*	0 ± 4	13 ± 2	0 ± 2	0 ± 2	0.277	0.312	0.465
Full recall (pts.)	18 ± 3	0 ± 4	0 ± 6	18 ± 1	0 ± 2	0 ± 1	0.709	0.576	0.373
**NCT**									
Numbers (sec)	45 ± 14	0 ± 16	−7 ± 17	44 ± 20	−1 ± 12	−4 ± 13	0.427	0.667	0.769
Numbers and letters (sec)	106 ± 47	0 ± 41	−2 ± 34	96 ± 34	−4 ± 28	−10 ± 38	0.331	0.671	0.664
**RWFT**									
Phonemes, 1st attempt (pts.)	15 ± 4	0 ± 4	0 ± 4	14 ± 4	0 ± 4	0 ± 4	0.260	0.596	0.883
Phonemes, 2nd attempt (pts.)	11 ± 3	1 ± 4*	0 ± 4	10 ± 4	0 ± 5	1 ± 5*	0.227	0.557	0.143
Semantic, 1st attempt (pts.)	18 ± 6	0 ± 8*	−3 ± 6*	20 ± 6	−2 ± 9	−3 ± 7*	0.377	0.753	0.160
Semantic, 2nd attempt (pts.)	13 ± 3	0 ± 4	0 ± 5	12 ± 4	1 ± 4	0 ± 5	0.268	0.940	0.897
**NRT**									
Ahead (pts.)	7 ± 1	0 ± 1	0 ± 1	7 ± 2	0 ± 1*	0 ± 1	0.798	0.661	0.728
Backwards (pts.)	5 ± 1	0 ± 1	0 ± 2	5 ± 1	0 ± 2*	0 ± 1	0.846	0.681	0.184
Total (pts.)	13 ± 2	0 ± 1	0 ± 2	13 ± 3	0 ± 2	0 ± 2	0.477	0.263	0.956

Results of the cognitive tests; patients ≥60 years of age; baseline values and changes over time; means and SD; within-group and between-group comparisons with Wilcoxon tests and Mann–Whitney U-tests; *p < 0.05; **p < 0.01. MMSE, mini-mental state examination; NCT, number-connection test; NRT, number-recall test; RCFT, Rey–Osterrieth complex Figure test; RWFT, Regensburg word fluency test; VLMT, verbal learning memory test. For MMSE, RCFT, VLMT, RWFT, and NRT, increases are improvements, while for the NCT, decreases indicate improvement.

Older patients receiving placebo improved more strongly in one VLMT score (after 1 year), one RWFT score, and the total NRT score (both after 2 years, only) compared to younger subjects. In the fiber group, one VLMT score improved significantly strongly among older patients compared to younger ones. However, despite statistical significance, all these differences were clinically irrelevant (Supplementary Tables 1, 2).

### Correlation of cognitive outcomes with age and metabolic parameters

To determine the overall sensitivity of the utilized memory tests to show age-dependent cognitive alterations in the context of impaired metabolism and lifestyle treatment, we investigated the correlations between age, fasting glucose, 2-h glucose, and HbA1c with all cognitive scores at baseline.

Herein, age was moderately correlated with weaker cognitive performance in the RCFT recall test, the VLMT, the NCT, and the backwards version of the NRT, i.e., the most challenging tests with the broadest range of individual results. Metabolic parameters only showed weak and spurious significant correlations with the VLMT recall interference list (fasting glucose and 2-h glucose) and one attempt of the RWFT (HbA1c) (Table 5). When correlating 1-year changes of glycemic and cognitive outcomes, no significant correlation was found for the placebo group, and only six spurious (out of 69 possible) correlations were found for the fiber group (data not shown).

**Table 5:** Correlation between cognitive scores, age, and major glycometabolic outcomes at baseline.

Cognitive test	Age		Fasting glucose		2-h glucose		HbA1c	
	*ρ*	*p*-value	*ρ*	*p* value	*ρ*	*p*-value	*ρ*	*p*-value
MMSE (pts.)	−0.003	0.966	−0.001	0.995	0.086	0.286	−0.091	0.249
RCFT								
Copying (pts.)	−0.027	0.718	0.025	0.761	0.081	0.312	0.042	0.600
Recall (pts.)	−0.258	<0.001***	0.083	0.304	−0.052	0.516	−0.023	0.769
VLMT								
1st attempt (pts.)	−0.252	<0.001***	−0.045	0.579	0.035	0.668	0.024	0.759
2nd attempt (pts.)	−0.291	<0.001***	−0.091	0.255	−0.096	0.231	0.010	0.896
3rd attempt (pts.)	−0.345	<0.001***	−0.030	0.713	−0.145	0.070	0.038	0.632
4th attempt (pts.)	−0.251	<0.001***	0.037	0.647	0.007	0.934	0.055	0.490
5th attempt (pts.)	−0.281	<0.001***	−0.020	0.808	−0.012	0.877	0.041	0.605
Interference list (pts.)	−0.275	<0.001***	−0.088	0.273	−0.077	0.339	0.120	0.130
6th attempt (pts.)	−0.311	<0.001***	0.013	0.869	−0.073	0.365	0.102	0.200
7th attempt (pts.)	−0.368	<0.001***	−0.042	0.599	−0.089	0.273	0.108	0.172
Recall list (pts.)	−0.327	<0.001***	0.023	0.774	0.038	0.638	0.096	0.228
Recall interference (pts.)	−0.237	0.002**	−0.174	0.029*	−0.224	0.005*	0.087	0.275
Full recall (pts.)	−0.172	0.022*	0.013	0.667	0.034	0.675	−0.012	0.877
NCT								
Numbers (sec)	0.366	<0.001***	−0.034	0.671	−0.038	0.639	−0.011	0.888
Numbers and letters (sec)	0.368	<0.001***	0.000	0.998	−0.092	0.251	0.090	0.257
RWFT								
Phonemes, 1st attempt (pts.)	0.028	0.711	0.066	0.414	0.036	0.653	−0.071	0.370
Phonemes, 2nd attempt (pts.)	0.020	0.788	0.113	0.161	0.022	0.785	−0.063	0.425
Semantic, 1st attempt (pts.)	−0.011	0.883	0.099	0.217	0.059	0.468	−0.219	0.005**
Semantic, 2nd attempt (pts.)	−0.004	0.955	0.006	0.939	−0.010	0.897	−0.045	0.572
NRT								
Ahead (pts.)	−0.051	0.505	0.025	0.753	−0.014	0.862	−0.070	0.375
Backwards (pts.)	−0.162	0.031*	0.087	0.278	0.021	0.792	0.025	0.752
Total (pts.)	−0.106	0.161	0.054	0.501	0.003	0.968	−0.046	0.562

Correlation analysis at baseline, rho (ρ) based on Spearman correlations *p < 0,05; **p < 0,01. MMSE, mini-mental state examination; NCT, number-connection test; NRT, number-recall test; RCFT, Rey–Osterrieth complex figure test; RWFT, Regensburg word fluency test; VLMT, verbal learning memory test.

## Discussion

Our present analysis of the Optimal Fiber Trial focused on cognitive outcomes, in a randomized, placebo-controlled dietary (2 years) and complex lifestyle intervention (1 year) in 180 participants with impaired glucose tolerance. We did not observe a statistically or clinically relevant impact of supplementation with insoluble cereal fiber on various aspects of memory, attention, and learning capabilities. In both the placebo and fiber groups, that is, the entire cohort that underwent a complex 1-year lifestyle program, we found minor improvements in verbal learning and short-term recall. These effects partially remained stable throughout the second year of intervention, while some cognitive scores deteriorated irrespective of age or treatment allocation. Overall, none of the between-group comparisons were strong enough to survive potential correction for multiple testing, which we abstained from for this exploratory analysis.

Cohort studies consistently assume a strong protective effect of high-fiber diets for cardiovascular diseases, affecting small and larger vessels, as well as complex age-related ailments, such as dementia. This *could* be related to fiber, but also to other concomitantly present nutrients in high-fiber diets, and even other factors besides diet (24–27). RCTs introducing specific complex dietary improvements containing high fiber loads inconsistently showed sustained memory functions, with benefits for the Mediterranean diet (24), but lack of effect for low-fat or MIND (23, 25–27). Weight-loss approaches delayed cognitive decline in some studies (28, 29, 32), but results are conflicting, with accelerated cognitive deterioration reported as well (62). RCTs on specific food components, such as micronutrients, failed to show a benefit when administered irrespective of baseline deficiency or when applied to patients with either fully normal or severely impaired cognitive function (63, 64). Previous studies on fiber-rich cereals or specific types of indigestible carbohydrates also inconsistently reported benefits from supplementation (33–45).

From that perspective, our own results fall in line with the literature. Isolated fiber intervention was ineffective compared to placebo, while the lifestyle program based on combined diet, exercise, and behavioral training provided small benefits at least for its duration, if not for another year of observational follow-up. Every healthy diet comes with a high amount of fiber and may improve metabolism, inflammation, and overall well-being. Fiber alone (be it every kind of fiber or specific types of fiber), however, might be a minor contributor or even just a bystander of other more relevant nutrients, such as polyphenols or vitamins. At least for some of these, mainly in the means of compensated malnutrition, RCTs reported successful supplementation for the sake of cognitive improvement (65–67). Specific foods with these components (not just the isolated substance), such as olive oil or green tea, might be effective as well (68, 69). Even for generally healthy, complex diets with a high load of fiber, low-fat and MIND (DASH) were ineffective with respect to cognitive outcomes, while the Mediterranean has proven benefits (25–27).

We are convinced that both our cohort and our methodological approach were suitable to show a clinically relevant effect on cognition, if it exists. First, an intervention duration of 2 years provides enough time for beneficial effects or natural decay to take place. Second, separating lifestyle intervention and supplementation after the first year allows for a more disentangled analysis, which of the interventions is effective. Third, the variety of cognitive tests was able to cover a wide range of cognitive functions both qualitatively and quantitatively in our cohort of middle-aged-to-old prediabetes patients with a broad span of test results. Most test scores correlated with age, even though they may not have been designed to evaluate memory functions in the elderly (such as the RCFT, which serves as a neglect test in neurology). While the MMSE was not able to highlight differences in cognitive abilities in our rather healthy sample in the sense of a ceiling effect, other tests, such as RCFT, NCT, and RWFT, were more challenging to the patients, providing more sensitivity for the detection of changes in memory functions. Fourth, our previous publications on OptiFiT clarify that mere supplementation was sufficient to affect metabolism, ergo, a specific interaction between fiber supplement and certain nutrients or the presence of a typical fiber-food-matrix was not required.

Surprisingly, cognitive test scores did not correlate with metabolic parameters. The relatively limited range of cognitive test scores in our cohort should not be causal for the absence of correlations, as such correlations were found with age for most of the tests. Rather more, the range of glycemic values might have been too small to show an impact of glycemia on cognition, both cross-sectionally and over time. Cognitive decline due to prediabetes is theoretically possible, but it happens very slowly. Two years of intervention and observation may have been too short to detect age-related decline and intervention-based conservation of memory functions. Patients with severely deteriorated glucose regulation were not included in the study or dropped out as soon as overt diabetes was diagnosed. Thus, data from patients with concomitant diabetes-related decline might have been censored. As the majority of diabetes diagnoses occurred at the last visit and ITT analysis was used, these methodological limitations should not explain the absence of effects of age and interventions. On the positive side, investigating the effects on patients with prediabetes reduces the chances of medication effects, as antidiabetic drugs are not involved; other pharmaceutical agents (such as antihypertensives, statins, and acetylsalicylic acid) are less commonly used compared to T2D patients; medication against dementia was not present among our patients’ prescriptions.

Several limitations should be considered when interpreting our findings. Parallel intervention with lifestyle program and supplementation might create overlapping effects, but discontinuation of PREDIAS after 12 months and consistent randomized supplementation should have allowed for an unmasked evaluation of both intervention effects. Theoretically, patients in the placebo group could have achieved a high fiber intake by plain dietary improvement. Our recently published data show that “supplementation only” led to relevantly increased fiber intake (47). This behavioral inertia is known from earlier studies (70). Moreover, apart from differences in fiber intake, intervention groups did not differ in moderate overall changes of diet, physical activity, and body weight.

RCTs on the cognitive effects of specific nutrients are rare and usually limited in statistical power. Our study outranks most of these trials in duration and sample size, although even larger and longer-term trials might be needed to detect small differences (65–69). As OptiFiT was powered for the primary metabolic outcome, not for the exploratory cognitive outcomes; beta errors might contribute to the absence of statistical significance. Given the huge set of parameters and time points, additional correction for multiple testing would have been applied in a non-exploratory analysis, further diminishing statistical significance without changing clinical relevance.

For mechanistic analyses, OptiFiT provides only a limited set of (already analyzed) samples to pinpoint relevant pathways of metabolic action that are truly involved in the health benefits of insoluble, poorly fermentable fiber. Stool samples were not collected, and incretins and SCFA were not measured. In the overall study cohort, inflammatory outcomes suggest that our fiber supplement had a moderate impact on leucocyte counts and CRP (but not IL-18) (47, 49).

Our dataset may have suffered from ceiling and bottom effects, as the investigated cohort may have been too healthy with respect to cognition. Baseline values, e.g., for the MMSE, indicated high cognitive capabilities, limiting the chance for improvement and restricting the potential outcome toward decline. At baseline, most of our tests did not show differences between younger and older participants and did not correlate with age. Our tests may therefore have been insensitive to changes in cognitive abilities despite the very long follow-up in this elderly cohort. Future studies should implement more sensitive instruments, such as the MoCA or ADAS-Cog (71, 72).

In summary, we report no consistent, statistically significant, and clinically relevant effects of a 2-year intervention with twice-daily supplements containing insoluble fiber in a cohort receiving a simultaneous first-year lifestyle guidance, on memory functions in elderly patients with prediabetes. Larger, long-term RCTs might be needed to detect effects on cognitive outcomes. Such studies should provide a sufficiently long follow-up to cover slowly progressing changes in cognition, hard clinical endpoints such as the onset of dementia, and assess biochemical parameters for parallel documentation of both cognitive health (e.g., BDNF, S100-beta, and inflammatory proteins) and mechanisms of fiber action (e.g., gut microbiome, SCFA levels, and bile acids).

## Data Availability

The raw data supporting the conclusions of this article will be made available by the authors upon specific request.
