# Supplementary material for: Effects of lifestyle intervention and supplementation with insoluble oat fiber on cognitive functions in patients with prediabetes: a secondary analysis of the Optimal Fiber Trial
Source: Front Nutr. 2026 Jan 16;12:1699958. doi: 10.3389/fnut.2025.1699958 (PMC12857309; doi:10.3389/fnut.2025.1699958)
Supplement: Supplementary file 1 [file Table_1.docx]

Suppl. table 1: Comparison of cognitive outcomes between younger and older subjects in the placebo group

| **Cognitive test** | **Younger subjects** | | | **Older subjects** | | | **P value** | | |
| --- | --- | --- | --- | --- | --- | --- | --- | --- | --- |
|  | **Baseline** | **1 year** | **2 years** | **Baseline** | **1 year** | **2 years** | **Baseline** | **1 year** | **2 years** |
| **MMSE** (pts.) | 28 ± 1 | 0 ± 1 | 0 ± 1 | 28 ± 1 | 0 ± 1 | 0 ± 1 | 0,721 | 0,994 | 0,735 |
| **RCFT** |  |  |  |  |  |  |  |  |  |
| Copying (pts.) | 35 ± 1 | 0 ± 1 | -1 ± 7 | 35 ± 1 | -1 ± 6 | 0 ± 2 | 0,978 | 0,632 | 0,285 |
| Recall (pts.) | 23 ± 5 | 1 ± 5 | 1 ± 4 | 20 ± 5 | 1 ± 6 | 0 ± 4 | **0,001** | 0,823 | 0,460 |
| **VLMT** |  |  |  |  |  |  |  |  |  |
| 1st attempt (pts.) | 6 ± 1 | 0 ± 1 | 1 ± 2 | 5 ± 1 | 0 ± 2 | 0 ± 2 | 0,272 | 0,440 | 0,968 |
| 2nd attempt (pts.) | 9 ± 1 | 0 ± 2 | **1 ± 2*** | 8 ± 2 | 0 ± 1 | **0 ± 2*** | **0,004** | 0,086 | 0,260 |
| 3rd attempt (pts.) | 10 ± 2 | 0 ± 3 | 1 ± 3 | 9 ± 2 | 0 ± 2 | 1 ± 2 | **0,013** | **0,040** | 0,185 |
| 4th attempt (pts.) | 11 ± 2 | 0 ± 3 | 0 ± 2 | 10 ± 2 | 0 ± 2 | 1 ± 2 | 0,065 | 0,902 | 0,656 |
| 5th attempt (pts.) | 12 ± 1 | 0 ± 3 | 0 ± 2 | 11 ± 2 | 0 ± 3 | **0 ± 2*** | **0,015** | 0,143 | 0,478 |
| Interference list (pts.) | 6 ± 2 | 0 ± 2 | 0 ± 2 | 5 ± 1 | 0 ± 1 | **0 ± 1*** | **0,034** | 0,861 | 0,473 |
| 6th attempt (pts.) | 10 ± 2 | **0 ± 2*** | 1 ± 2 | 9 ± 3 | 0 ± 2 | 0 ± 2 | 0,068 | 0,787 | 0,552 |
| 7th attempt (pts.) | 10 ± 3 | 0 ± 2 | 0 ± 3 | 9 ± 3 | 0 ± 3 | 0 ± 3 | **0,044** | 0,478 | 0,501 |
| Recall list (pts.) | 13 ± 2 | 0 ± 2 | 0 ± 4 | 12 ± 1 | **0 ± 2*** | 0 ± 1 | **0,005** | 0,567 | 0,739 |
| Recall interference (pts.) | 14 ± 2 | 0 ± 2 | 0 ± 3 | 13 ± 2 | 0 ± 2 | 0 ± 2 | **0,014** | 0,702 | 0,140 |
| Full recall (pts.) | 19 ± 3 | 0 ± 3 | 0 ± 4 | 18 ± 1 | 0 ± 2 | 0 ± 1 | 0,081 | 0,728 | 0,368 |
| **NCT** |  |  |  |  |  |  |  |  |  |
| numbers (sec) | 34 ± 18 | -4 ± 21 | **-5 ± 27*** | 44 ± 20 | -1 ± 12 | -4 ± 13 | **0,001** | 0,530 | 0,617 |
| numbers and letters (sec) | 70 ± 31 | -5 ± 45 | -5 ± 23 | 96 ± 34 | -4 ± 28 | -10 ± 38 | **0,001** | 0,454 | 0,841 |
| **RWFT** |  |  |  |  |  |  |  |  |  |
| Phonemes, 1st attempt (pts.) | 15 ± 4 | 0 ± 4 | 0 ± 4 | 14 ± 4 | 0 ± 4 | 0 ± 4 | 0,222 | 0,356 | 0,936 |
| Phonemes, 2nd attempt (pts.) | 12 ± 4 | 0 ± 5 | 1 ± 4 | 10 ± 4 | 0 ± 5 | **1 ± 5*** | 0,311 | 0,881 | **0,026** |
| Semantic, 1st attempt (pts.) | 20 ± 7 | 1 ± 9 | **-4 ± 4*** | 20 ± 6 | -2 ± 9 | **-3 ± 7*** | 0,939 | 0,208 | 0,709 |
| Semantic, 2nd attempt (pts.) | 13 ± 4 | 1 ± 4 | **1 ± 3*** | 12 ± 4 | 1 ± 4 | 0 ± 5 | 0,189 | 0,618 | 0,663 |
| **NRT** |  |  |  |  |  |  |  |  |  |
| ahead (pts,) | 7 ± 2 | 0 ± 1 | 0 ± 1 | 7 ± 2 | **0 ± 1*** | 0 ± 1 | 0,899 | 0,300 | 0,284 |
| backwards (pts.) | 6 ± 1 | 0 ± 1 | 0 ± 2 | 5 ± 1 | **0 ± 2*** | 0 ± 1 | **0,011** | 0,174 | 0,254 |
| total (pts.) | 14 ± 3 | 0 ± 2 | 0 ± 2 | 13 ± 3 | 0 ± 2 | 0 ± 2 | 0,157 | 0,592 | **0,009** |

Legend: results of the cognitive tests comparing age groups in the placebo arm; baseline values and changes over time; younger patients are at <60 years of age; older patients are ≥ 60 years of age; means and SD; within-group comparisons with Wilcoxon-tests; between-group comparisons with Mann-Whitney-U-tests; *: p<0,05; **: p<0,01. MMSE: mini-mental state examination; NCT: number-connection test; NRT: number-recall test, RCFT: Rey-Osterrieth Complex Figure test, RWFT: Regensburg word fluency test; VLMT: verbal learning memory test. For MMSE, RCFT, VLMT, RWFT and NRT, increases are improvements, while for the NCT, decreases indicate improvement.
