# Supplementary material for: Effects of lifestyle intervention and supplementation with insoluble oat fiber on cognitive functions in patients with prediabetes: a secondary analysis of the Optimal Fiber Trial
Source: Front Nutr. 2026 Jan 16;12:1699958. doi: 10.3389/fnut.2025.1699958 (PMC12857309; doi:10.3389/fnut.2025.1699958)
Supplement: Supplementary file 2 [file Table_2.docx]

Suppl. table 2: Comparison of cognitive outcomes between younger and older subjects in the fiber group

| **Cognitive test** | **Younger subjects** | | | **Older subjects** | | | **P value** | | |
| --- | --- | --- | --- | --- | --- | --- | --- | --- | --- |
|  | **Baseline** | **1 year** | **2 years** | **Baseline** | **1 year** | **2 years** | **Baseline** | **1 year** | **2 years** |
| **MMSE** (pts.) | 28 ± 1 | 0 ± 1 | 0 ± 1 | 28 ± 1 | 0 ± 1 | 0 ± 1 | 0,767 | 0,794 | 0,189 |
| **RCFT** |  |  |  |  |  |  |  |  |  |
| Copying (pts.) | 35 ± 1 | 0 ± 1 | 0 ± 7 | 35 ± 1 | **0 ± 2*** | **-1 ± 2*** | 0,858 | 0,250 | 0,592 |
| Recall (pts.) | 19 ± 6 | 1 ± 7 | **5 ± 6**** | 18 ± 6 | **2 ± 4**** | 1 ± 6 | 0,161 | 0,073 | 0,445 |
| **VLMT** |  |  |  |  |  |  |  |  |  |
| 1st attempt (pts.) | 6 ± 1 | 0 ± 1 | 0 ± 1 | 5 ± 2 | **1 ± 2*** | **0 ± 2*** | 0,067 | 0,068 | 0,770 |
| 2nd attempt (pts.) | 9 ± 2 | **1 ± 2*** | **1 ± 2*** | 8 ± 2 | **1 ± 2**** | **0 ± 2**** | 0,196 | 0,693 | 0,111 |
| 3rd attempt (pts.) | 10 ± 1 | **1 ± 1**** | **1 ± 1**** | 10 ± 2 | 0 ± 2 | 0 ± 2 | 0,074 | 0,356 | 0,846 |
| 4th attempt (pts.) | 11 ± 2 | **1 ± 3**** | **1 ± 3**** | 10 ± 2 | 0 ± 2 | 0 ± 2 | 0,233 | 0,743 | 0,442 |
| 5th attempt (pts.) | 11 ± 2 | 0 ± 2 | 1 ± 3 | 10 ± 3 | 0 ± 2 | 0 ± 2 | **0,050** | 0,312 | 0,426 |
| Interference list (pts.) | 6 ± 2 | 0 ± 2 | 0 ± 1 | 5 ± 2 | **0 ± 1**** | **0 ± 1**** | 0,088 | 0,053 | 0,994 |
| 6th attempt (pts.) | 10 ± 3 | 0 ± 2 | 0 ± 2 | 8 ± 2 | **1 ± 2**** | 0 ± 2 | **0,001** | 0,165 | 0,693 |
| 7th attempt (pts.) | 11 ± 2 | **0 ± 1*** | 0 ± 2 | 8 ± 2 | **1 ± 2**** | 0 ± 2 | **0,001** | 0,671 | 0,409 |
| Recall list (pts.) | 13 ± 1 | 0 ± 1 | 0 ± 1 | 13 ± 2 | 0 ± 2 | 0 ± 3 | **0,012** | **0,036** | 0,180 |
| Recall interference (pts.) | 14 ± 1 | 0 ± 2 | 0 ± 1 | 13 ± 2 | **1 ± 3*** | 0 ± 4 | 0,110 | 0,728 | 0,565 |
| Full recall (pts.) | 19 ± 0 | 0 ± 1 | 0 ± 0 | 18 ± 3 | 0 ± 4 | 0 ± 6 | 0,179 | 0,897 | 0,334 |
| **NCT** |  |  |  |  |  |  |  |  |  |
| numbers (sec) | 36 ± 10 | 0 ± 16 | -3 ± 9 | 45 ± 14 | 0 ± 16 | -7 ± 17 | **0,002** | 0,549 | 0,448 |
| numbers and letters (sec) | 78 ± 28 | 2 ± 32 | 1 ± 37 | 106 ± 47 | 0 ± 41 | -2 ± 34 | **0,002** | 0,841 | 0,613 |
| **RWFT** |  |  |  |  |  |  |  |  |  |
| Phonemes, 1st attempt (pts.) | 14 ± 5 | 1 ± 4 | 0 ± 4 | 15 ± 4 | 0 ± 4 | 0 ± 4 | 0,362 | 0,403 | 0,309 |
| Phonemes, 2nd attempt (pts.) | 10 ± 4 | **1 ± 3*** | **1 ± 3*** | 11 ± 3 | **1 ± 4*** | 0 ± 4 | 0,217 | 0,449 | 0,810 |
| Semantic, 1st attempt (pts.) | 19 ± 5 | 0 ± 8 | 0 ± 6 | 18 ± 6 | **0 ± 8*** | **-3 ± 6*** | 0,493 | 0,300 | 0,596 |
| Semantic, 2nd attempt (pts.) | 12 ± 4 | 0 ± 3 | 0 ± 3 | 13 ± 3 | 0 ± 4 | 0 ± 5 | 0,270 | 0,268 | 0,308 |
| **NRT** |  |  |  |  |  |  |  |  |  |
| ahead (pts,) | 7 ± 1 | 0 ± 1 | 0 ± 1 | 7 ± 1 | 0 ± 1 | 0 ± 1 | 0,916 | 0,158 | 0,459 |
| backwards (pts.) | 5 ± 1 | 0 ± 1 | 0 ± 1 | 5 ± 1 | 0 ± 1 | 0 ± 2 | 0,439 | 0,947 | 0,573 |
| total (pts.) | 13 ± 3 | 0 ± 1 | 0 ± 2 | 13 ± 2 | 0 ± 1 | 0 ± 2 | 0,862 | 0,643 | 0,596 |

Legend: results of the cognitive tests comparing age groups in the fiber arm; baseline values and changes over time; younger patients are at <60 years of age; older patients are ≥ 60 years of age; means and SD; within-group comparisons with Wilcoxon-tests; between-group comparisons with Mann-Whitney-U-tests; *: p<0,05; **: p<0,01. MMSE: mini-mental state examination; NCT: number-connection test; NRT: number-recall test, RCFT: Rey-Osterrieth Complex Figure test, RWFT: Regensburg word fluency test; VLMT: verbal learning memory test. For MMSE, RCFT, VLMT, RWFT and NRT, increases are improvements, while for the NCT, decreases indicate improvement.
